# Cellular Mechanisms of Human Atherogenesis: Focus on Chronification of Inflammation and Mitochondrial Mutations

**DOI:** 10.3389/fphar.2020.00642

**Published:** 2020-05-14

**Authors:** Alexander M. Markin, Igor A. Sobenin, Andrey V. Grechko, Dongwei Zhang, Alexander N. Orekhov

**Affiliations:** ^1^Laboratory of Infection Pathology and Molecular Microecology, Institute of Human Morphology, Moscow, Russia; ^2^Laboratory of Medical Genetics, Institute of Experimental Cardiology, National Medical Research Center of Cardiology, Moscow, Russia; ^3^Laboratory of Angiopathology, Institute of General Pathology and Pathophysiology, Moscow, Russia; ^4^Federal Research and Clinical Center of Intensive Care Medicine and Rehabilitology, Moscow, Russia; ^5^Diabetes Research Centre, Traditional Chinese Medicine School, Beijing University of Chinese Medicine, Beijing, China

**Keywords:** atherosclerosis, nuclear genome, mitochondria, mutations, inflammation, mtDNA

## Abstract

Atherosclerosis is one of the most common diseases of the cardiovascular system that leads to the development of life-threatening conditions, such as heart attack and stroke. Arthrosclerosis affects various arteries in the human body, but is especially dangerous in the arteries alimenting heart and brain, aorta, and arteries of the lower limbs. By its pathophysiology, atherosclerosis is an inflammatory disease. During the pathological process, lesions of arterial intima in the form of focal thickening are observed, which form atherosclerotic plaques as the disease progresses further. Given the significance of atherosclerosis for the global health, the search for novel effective therapies is highly prioritized. However, despite the constant progress, our understanding of the mechanisms of atherogenesis is still incomplete. One of the remaining puzzles in atherosclerosis development is the focal distribution of atherosclerotic lesions in the arterial wall. It implies the existence of certain mosaicism within the tissue, with some areas more susceptible to disease development than others, which may prove to be important for novel therapy development. There are many hypotheses explaining this phenomenon, for example, the influence of viruses, and the spread in the endothelium of the vessel multinucleated giant endothelial cells. We suggest the local variations of the mitochondrial genome as a possible explanation of this mosaicism. In this review, we discuss the role of genetic variations in the nuclear and mitochondrial genomes that influence the development of atherosclerosis. Changes in the mitochondrial and nuclear genome have been identified as independent factors for the development of the disease, as well as potential diagnostic markers.

## Introduction

Atherosclerosis is a multifactorial disease with a complex pathogenesis. Multiple factors were shown to be involved in atherosclerotic lesion formation, and many knowledge gaps still remain. Atherosclerotic lesions can develop in any artery, but are especially dangerous in large vessels that aliment the heart, brain, and other vital organs. Macroscopically, the lesions are seen as local accumulation of fat in the arterial wall (so-called fatty streak), which is followed by local thickening of the innermost arterial wall layer called intima. At the advanced stages, the plaques acquire a thick fibrous cap, which separates them from the vessel lumen and circulating blood. Currently, several key factors in atherosclerotic lesion formation are considered. There is no doubt that impaired endothelial function and increased permeability plays an important role, especially at the initial stages of the lesion development (Mundi et al., 2018). Alterations of lipid metabolism and lipoprotein modifications are prerequisites of lipid accumulation (Summerhill et al., 2019). Chronic inflammation and immune disorders have been extensively studied in relation to atherosclerosis, which is currently regarded as an inflammatory disease (Frostegård, 2013; Rea et al., 2018). During the recent years, the prominent role of genetic factors in atherosclerosis development has been recognized, and numerous identified genes opened new possibilities for novel therapies development (Martínez et al., 2017; Wang W. et al., 2019; González-Becerra et al., 2019; Jha et al., 2019).

Numerous genetic studies were conducted to establish risk factors for the development of atherosclerosis (Björnsson et al., 2019; Li et al., 2019; Rincón et al., 2019). Researchers are constantly discovering new mutations specifically associated with cardiovascular disease. Apart from nuclear genome, mitochondrial genome variants may also be important as risk factors and disease modifiers. It is possible that mitochondrial DNA (mtDNA) mutations are responsible for predisposition to the development of atherosclerotic lesions (Sinyov et al., 2017). Mitochondria are semi-autonomous organelles that bear the genes of many, but not all mitochondrial proteins in their circular genome that resembles a bacterial chromosome. Mitochondrial dysfunction affects the cellular energy balance, metabolism and survival, and results in the development of mitochondrial cytopathies. These diseases can be caused both by nuclear genes encoding mitochondrial proteins or by mtDNA mutations that affect either mitochondrial proteins or transport RNA (tRNA). In this review, we will summarize the current knowledge on mtDNA mutations and mitochondrial dysfunction as pathophysiological factors of atherosclerosis development.

## Cellular Mechanisms of Atherogenesis

Atherosclerotic lesion development takes place in the intimal layer of the arterial wall. Adult intima is a rather thick formation with complex architecture and heterogeneous cellular composition. The intima is separated from the lumen of the vessel by a monolayer of endothelial cells. Endothelium plays a key role in the transport of cells and non-cellular components of blood from the arterial bed to the vascular wall (Krüger-Genge et al., 2019). Endothelial lining is heterogeneous (Romanov et al., 1995). In addition to cells of normal shape and size, clusters of giant multinucleated endothelial cells are found in the endothelial monolayer. These giant multinucleated cells appear only during the life period, in which atherosclerosis development is most frequent, and are typically not present in young individuals. Their appearance is important for understanding the mechanisms of atherogenesis, since clusters of such cells appear to be more common in the areas predisposed to atherosclerosis. Usually these are hemodynamic stress zones (for example, bifurcation and branching of blood vessels). Presence of such zones may partially explain the mosaicism of atherosclerosis. Lesions are not diffuse, but occur locally or focally, which may be associated with a local disturbance in the permeability of giant multinucleated endothelial cells.

Under the basal membrane, intima is populated by different types of cells (Rekhter et al., 1991). Immune cells such as macrophages (3–5%), dendritic cells (0.3%), and others, are located near the endothelium. Deeper layers contain elongated smooth muscle cells (70%) and pericytes or pericyte-like cells (25–30%) (Orekhov et al., 2014). Pericyte-like cells have a stellate shape and are connected through gap junctions with each other, forming a three-dimensional network, which serves as a kind of second line of immune defense (Ivanova et al., 2015). Pericyte-like cells can perform the functions of phagocytes, are able to secrete pro-inflammatory cytokines (Hill et al., 2014), and can also act as antigen-presenting cells (Ivanova and Orekhov, 2016a). In stimulating the immune response, pericytes are inferior in effectiveness to “professional” immune cells, but due to their abundancy in the arterial wall they are able to actively participate in the innate immunity reactions.

Cellular composition of the arterial wall undergoes profound changes in atherosclerotic lesion areas (Stary, 1990; Xu et al., 1990; Padarti and Zhang, 2018). Lesion development is accompanied by a local increase of the number of cells (cellularity), especially the numbers of macrophages and hematogenous cells. Moreover, the cellular network of pericyte-like cells disintegrates, with loss of intercellular communication and changes of cellular phenotype. Pericyte-like cells, macrophages, and some smooth muscle cells accumulate lipids, turning into foam cells, which have a distinctive appearance due to the lipid droplets accumulating in their cytoplasm. Presence of such cells in the subendothelial space of the arterial wall is a known early manifestation of atherosclerosis.

The primary source of lipids that accumulate in foam cells is atherogenic modified low density lipoprotein (LDL). Particles of LDL circulate in the blood and undergo chemical modifications affecting the glycoconjugate, lipid, and protein moieties (Ivanova et al., 2017; Orekhov and Myasoedova, 2019). Noteworthy, it is the multiply modified LDL that was found in the circulation of atherosclerotic patients, and not specific LDL modifications that have been characterized in *in vitro* experiments, such as oxidized LDL. Atherogenic modification of LDL begins with desialylation, and continues with changes of the lipoprotein particle size, density, and electric charge resulting in the formation of small dense and electronegative LDL fraction. Oxidation is likely to occur at later stages of atherogenic LDL modification. LDL atherogenicity is enhanced by the formation of self-associates and circulating immune complexes containing modified LDL and anti-LDL autoantibodies (Kacharava et al., 1993).

Native (unmodified) LDL does not cause the accumulation of intracellular lipids in cultured cells, because LDL binds to a specialized LDL receptor (LDLR), and undergoes degradation through the receptor-mediated pathway (Zhang et al., 2016). The excess lipids are eliminated from the cell due to the efflux, in which high-density lipoprotein (HDL) and transporter proteins play a key role (Laatsch et al., 2012).

Associates of modified LDL stimulate the phagocytic activity of subendothelial macrophages and pericytes. Following phagocytosis, inflammatory cytokines are secreted, which attracts monocytes and other immune cells to the emerging site of inflammation. Inflammatory cytokines contribute to further accumulation of intracellular lipids induced by modified atherogenic LDL. Moreover, in some cases, lipid accumulation induction is observed even in absence of modified LDL (**Table 1**).

**Table 1:** The effect of IL-6, IL-8, IL-12, IL-15, IL-17, IL-18 on the concentration of cholesterol in THP-1 cells.

Comparison groups		Relative cell cholesterol concentration, % (SD, %)	P (t-test)		P (M-W)	
			vs control	vs LDL	vs control	vs LDL
1	Control	100.0 (20.4)	–	–	–	–
2	LDL	164.1 (39.4)	<0.001	–	<0.001	–
3	LDL+IL-6	174.8 (28.0)	<0.001	0.134	<0.001	0.121
4	IL-6	116.5 (20.9)	<0.001	<0.001	<0.001	<0.001
5	LDL+IL-8	246.8 (26.5)	<0.001	<0.001	<0.001	<0.001
6	IL-8	117.0 (21.9)	0.012	0.001	0.035	0.001
7	LDL+IL12	180.2 (26.5)	<0.001	0.258	<0.001	0.27
8	IL-12	93.0 (18.5)	0.285	<0.001	0.395	<0.001
9	LDL+IL15	175.8 (23.6)	<0.001	0.05	<0.001	0.096
10	IL-15	100.3 (10.0)	0.943	<0.001	0.631	<0.001
11	LDL+IL17	107.0 (33.8)	0.586	<0.001	0.861	0.001
12	IL17	83.4 (29.7)	0.159	<0.001	0.201	<0.001
13	LDL+IL18	114.1 (76.0)	0.615	0.002	0.948	0.003
14	IL18	100.7 (42.0)	0.967	<0.001	0.928	<0.001

The study was performed on the THP-1 human cell culture obtained from American Type Culture collection (ATCC). Cells were maintained in RPMI with 10% heat-inactivated fetal bovine serum, 2 mM L-glutamine and 100 μg/ml penicillin/streptomycin. THP-1 monocytes were differentiated into macrophage-like cells by incubation for 3 days in medium supplemented with phorbol 12-myristate 13-acetate (PMA) (50 ng/ml). Total LDL (density 1.019–1.063 g/ml) were isolated from hyperlipidemic plasma of donors by preparative ultracentrifugation as previously described (Chapman et al., 1981). After a 3-day incubation, both LDL (100 μg/ml) and interleukins (Il6 and Il15 each at a concentration of 50 ng/ml) were added simultaneously and incubated for 24 h. After incubation lipids were isolated using the Folch method (Folch et al., 1957), and cholesterol was quantified as previously described (Gamble et al., 1978). Protein was measured in 40 µl aliquots of cell lysate using Lowry method (Lowry et al., 1951) with bovine serum albumin solution as a standard. All measurements were performed in duplicate.

**Figure 1:**
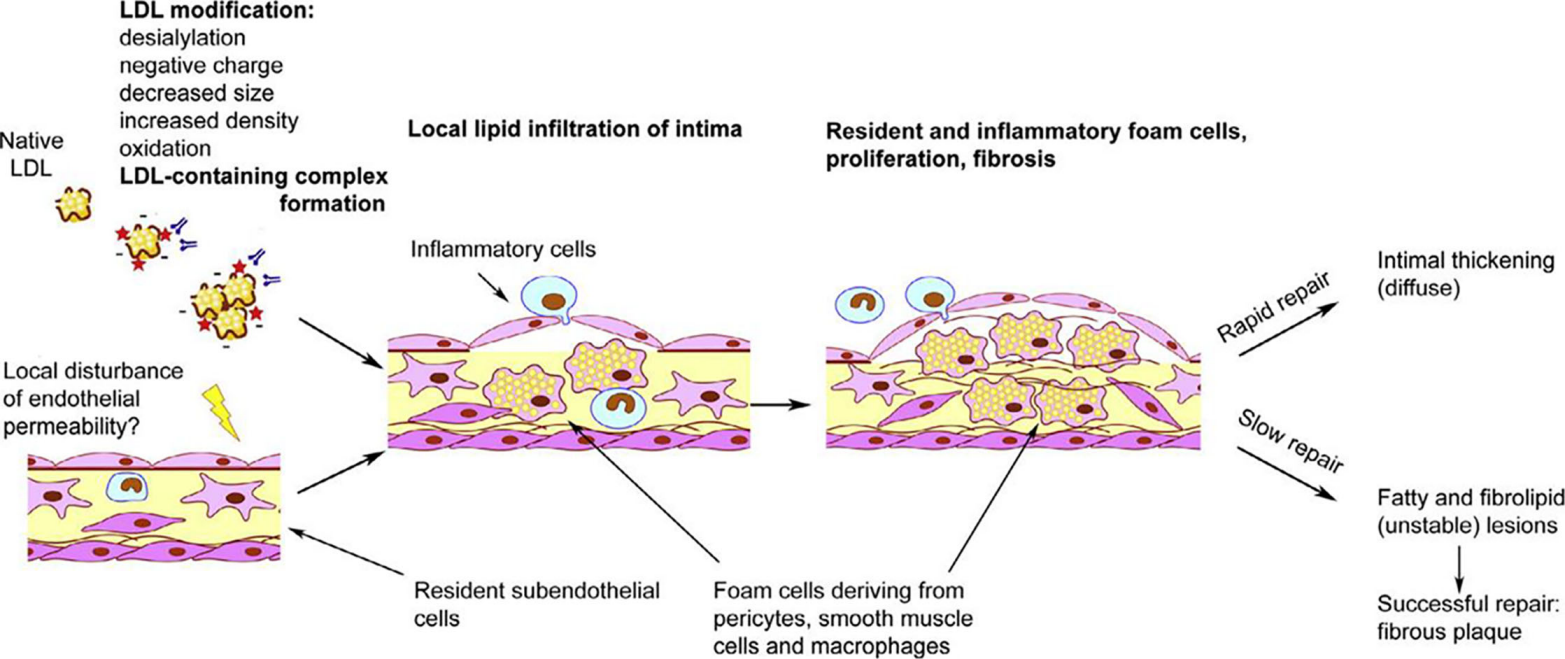
Schematic overview of initiation of atherosclerotic lesion formation. From Orekhov and Ivanova (2016), with permission.

Therefore, not the accumulation of intracellular lipids caused by LDL, but the immune response to the interaction of the cell with LDL promotes or even induces the formation of foam cells. Intracellular lipid accumulation leads to the rupture of cell contacts in the three-dimensional network of pericyte-like cells (Ivanova et al., 2015). This is accompanied by increased proliferative activity and stimulation of extracellular matrix synthesis (Ponticos and Smith, 2014; Orekhov et al., 2016). Such processes are characteristic of the reparative phase of the inflammatory reaction. Normally, only a small thickening of the intimal tissue remains at the site of inflammation (**Figure 1**) (Orekhov and Ivanova, 2016). This process may occur in the arteries without causing visible symptoms and lesions can accumulate over time, so that focal formations become a diffuse thickening. Diffuse intimate thickening is not an atherosclerotic lesion, but is considered normal for the arteries of an adult body (Nakashima et al., 2008; Subbotin, 2016).

According to current consensus, the primary event in atherosclerotic lesion development is local endothelial activation and increased permeability, which may be caused by hemodynamic forces occurring at the sites of blood vessel bends and bifurcations. Other known triggering factors of atherosclerosis include circulating mediators of inflammation and vasoactive substances, diet-induced alterations of the levels of circulating lipids, modified LDL, increased blood glucose level, and cigarette smoke. At the sites of local activation, the endothelial cells change their expression pattern, beginning to express cytokines and chemokines (interleukin 1 [IL-1]), tumor necrosis factor alpha (TNF-α), chemokines monocyte chemoattractant factor 1 (MCP-1), growth factors, such as platelet-derived growth factor (PDGF) and basic fibroblast growth factor (bFGF), and adhesion molecules. This leads to the increased recruitment and adhesion of circulating immune cells that interact with the endothelium and penetrate into the subendothelial space. During this process, circulating monocytes become activated and differentiate into macrophages. This further increases the pro-inflammatory signaling at the emerging lesion site. Macrophages are specialized phagocytes that actively participate in lipid uptake within the lesion and are an important source of foam cells (Koenig, 1999).

An increase in the concentration of pro-inflammatory cytokines leads to endoplasmic reticulum stress in the arterial wall cells, which in turn can lead to the initiation of apoptosis (Ivanova and Orekhov, 2016b). Cytokine-induced inflammation disrupts the normal functioning of mitochondria, their synthesis and mitophagy (Gkikas et al., 2018), which also leads to apoptosis. These effects contribute to the prolonged circulation of pro-inflammatory agents in the vascular bed, which can further stimulate the launch of a cascade of inflammatory reactions at the site of vascular damage.

It is plausible that atherosclerotic lesion development occurs when the inflammatory process cannot be resolved in a regular way and becomes chronic (**Figure 1**) (Orekhov and Ivanova, 2016). Thus, the response of the innate immunity is a trigger for the formation of foam cells, while violation of the normal immune response is the cause of inflammation chronification, which leads to the development of atherosclerotic lesions. It would be interesting to identify the genetic factors that regulate these processes.

## Variants of the Nuclear Genome Associated with Atherosclerosis

It is generally believed that in rare cases, single nucleotide substitute in nuclear genome may greatly affect the clinical phenotype. On the opposite, as a rule, it is impossible to establish a causal relationship between a certain variant of the nuclear genome and a phenotypic manifestation of this change. This is also true for detecting genetic predisposition to chronic pathologies like atherosclerosis and related cardiovascular diseases. In the last decade, the genome-wide associations study (GWAS) approach came into play and was widely applied. In general, this is a targeted search for associations between genomic variants and phenotypic traits, i.e. for the search of associations between single nucleotide polymorphisms (SNPs) and diseases. Such studies provide a great bulk of information, which, however, can hardly be implemented into practical healthcare. On the other hand, systematic approach to the search for such associations is necessary to assess the quality of the results of numerous studies in this area and to identify the most promising sets of markers for further detailed research (Belsky et al., 2013; den Hoed et al., 2015).

Noteworthy, GWAS data are not particularly valuable without modern computational methods. An effective tool for finding the dependencies between mutations and their manifestations are systems biology methods (Huan et al., 2013; Frades et al., 2019). Analysis of molecular networks allows explaining how genetic disorders interact with the environmental factors and why this interaction manifests itself in the form of a pathological phenotype. As an example, Talukdar et al. have analyzed the genetic data and gene expression profiles of several tissues (atherosclerotic arterial wall, internal mammary artery, liver, skeletal muscle, visceral fat, subcutaneous fat, and whole blood from the late-stage patients with coronary artery disease [CAD]). As the result, 30 regulatory gene networks (RGNs) were identified being associated with CAD development and their key mechanisms of action. As a proof of concept, the researchers aimed at key drivers (*AIP, DRAP1, POLR2I*, and *PQBP1* genes) in the RGN of the arterial wall with cross-species verification, including RNA processor genes. This RGN was re-identified in THP-1 foam cells and independently in blood-derived monocytes of CAD patients and macrophages from carotid atherosclerotic lesions. Such studies can help to better identify the candidate genes critical for the development of CAD identified in the framework of GWAS, and this is only the beginning of the path to achieving the goals of personalized medicine (Talukdar et al., 2016).

Currently, about 60 genomic regions associated with coronary heart disease have been identified. However, most cases of hereditary predisposition to atherosclerosis still cannot be explained. Thus, there exists a need for search of other loci associated with atherogenesis. An effective strategy can be a large-scale assessment of promising genomic associations, proposed as a part of various genome research projects. For example, some of the mutation changes detected are associated with endothelial cell dysfunction. Furthermore, genes regulating cellular adhesion, leucocyte migration, coagulation, inflammation, vascular smooth muscle cell differentiation and genes that regulate energy metabolism may prove to be relevant for atherosclerosis. A correlation analysis was performed that linked the identified gene regions with cell type-specific gene expression patterns and phenotypic features such as plasma protein levels (CARDIoGRAMplusC4D Consortium et al., 2013; Howson et al., 2017). Such studies may provide novel and important information that will help developing new therapies.

Numerous genetic studies have been conducted to establish the risk factors of atherosclerosis (Björnsson et al., 2019; Li et al., 2019b Rincón et al., 2019). For example, four new SNPs have recently been found recently to be specifically associated with abdominal aortic aneurysm (Marsman et al., 2019). There is evidence that an increased risk of cardiovascular disease (CVD) exists among bearers of haplogroup I1 (Y-DNA). The Y chromosome of these individuals was enriched in regulatory chromatin variants related to the development of coronary heart disease (Eales et al., 2019; Lusis, 2019). Aortic aneurysm as a whole is characterized by a pathogenesis similar to atherosclerosis, namely: infiltration by the inflammatory cells of the vessel wall, degradation of the extracellular matrix, and dysfunction of vascular smooth muscle cells (Wang Y. et al., 2019).

One way to control the CVD complications is to reduce the intake of saturated fats from food. However, if SNPs in the genes responsible for cholesterol metabolism are present, high levels of total cholesterol and LDL cholesterol and triglyceride, and low HDL cholesterol persist regardless of the diet (Walker et al., 2011).

In addition to mutations in coding regions of genes, changes in the regulatory regions are also capable of affecting gene expression. The long non-coding RNAs (lncRNAs), that are longer than 200 nucleotides, can regulate the expression of neighboring genes. Recent evidence suggests the role of lncRNAs expression on proliferation of vascular smooth muscle cells and apoptosis, and, consequently, on the risk of aortic aneurysm and atherosclerosis (Brozovich et al., 2016). The likely mechanism of such influence is overexpression of lncRNA-p21 and the increase of p53 downstream target genes *Puma*, *Bax*, *Noxa*, and *MDM2* at mRNA and protein level. This is consistent with the involvement of these genes in the regulation of cell survival, apoptosis, and proliferation (Wu Y. et al., 2014). Importantly, the expression of this lncRNA is suppressed in atherosclerotic plaques in *apoE^-/-^* mice. In addition, several SNPs at the 9p21 locus that are known to be associated with atherosclerosis were shown to have an impact on the expression of lncRNA ANRIL, which contains at least 21 exons and has multiple linear and circular isoforms. Polymorphisms within ANRIL were shown to be associated with increased risk of different types of cancer and atherosclerosis, obesity, and type 2 diabetes (Kong et al., 2018).

One of the genes implicated in aneurism formation is *LOX*, which encodes for lysyl oxidase, a protein responsible for cross-linking of collagen and elastin molecules during the formation of regular collagen fibers. *LOX* is important for mechanical integrity of the arterial wall, and missense mutations in this gene were shown to cause aneurysm formation and aortic dissection due to insufficient crosslinking of elastin and collagen in the aortic wall. The study that linked *LOX* to aneurism formation offered an algorithm that could be used to search mutational changes associated with the development of coronary heart disease (Lee et al., 2016).

In most cases, familial hypercholesterolemia develops in the presence of pathogenic variants of genes encoding the low-density lipoprotein receptor (*LDLR*), its ligand apolipoprotein B (*APOB*), or the proprotein convertase subtilisin/kexin type 9 (*PCSK9*), which takes part in the regulation of cholesterol metabolism. Binding of PCSK9 to EGF-A (extra-cellular domain of LDLR) leads to degradation of the receptor. A decrease in LDLR causes a decreased LDL metabolism, which can lead to hypercholesterolemia. The PCSK9 is synthesized in a soluble inactive form of proenzyme, which can be activated spontaneously during intramolecular processing in the endoplasmic reticulum (Gu et al., 2013; Cariou and Dijk, 2020). Other identified genes that were less frequently detected in familial hypercholesterolemia encode apolipoprotein E (*APOE*) and the signal-transducing adaptor family member 1 (*STAP1*) (Fouchier et al., 2014). The modern classification of lipid metabolism disorders was developed by Donald Fredrickson in 1965 and is based on changes in the profile of plasma lipoproteins. This classification was adopted by the World Health Organization, although it does not take into account the level of HDL, an important factor that reduces the risk of developing atherosclerosis, as well as the role of genes that cause lipid disorders (Di Taranto et al., 2019; Gomez et al., 2019). However, the genetic disorders described above are generally not specific for typical vascular wall cells, but appear to be important at the level of the whole organism.

## Variants of Mitochondrial Genome Associated With Atherosclerosis

Accumulating evidence indicates that mtDNA mutations play a role in atherosclerosis development alongside with nuclear genome polymorphisms. An important part of mitochondrial proteins that are indispensable for proper functioning of the respiratory chain and energy production are encoded by mtDNA. Therefore, mtDNA mutations can lead to serious consequences, such as altered energy homeostasis, impaired glucose and fat metabolism, elevated oxidative stress and, ultimately, cell damage and death. These processes are tightly implicated in atherosclerotic lesion development (Weakley et al., 2010).

The main function of mitochondria in the eukaryotic cell is oxidation of organic compounds and the use of the released energy for ATP synthesis and thermogenesis (Hu and Liu, 2011). The size of mtDNA, a double-stranded circular molecule, in human cells is about 16,600 nucleotide pairs. It encodes 2 rRNA, 22 tRNA, and 13 subunits of respiratory chain enzymes. Each mitochondrion can contain two to ten copies of mtDNA. If all mtDNA copies contain the same polymorphism, it is called homoplasmic, while presence of mutant and wild-type copies of the mitochondrial gene within the same cell is referred to as heteroplasmy. Mutations in mtDNA are responsible for a number of inherited human diseases. These changes are inherited almost exclusively through the maternal line (Chan, 2006; Sobenin et al., 2013; Tang et al., 2014; Strassheim et al., 2018).

Mitochondrial reactive oxygen species (ROS) generated by dysfunctional mitochondria not only contribute to cell damage during oxidative stress, but also act as intermediate signals, which are modulators of gene expression associated with the development of atherosclerosis. Mutational changes in the mitochondrial genome can partially explain the focal nature of the vascular wall damage in atherosclerosis. Cells of different sections of the vascular wall can vary significantly by the level of heteroplasmy, which leads to differences in cellular metabolism. Therefore, due to the development of mitochondrial dysfunction, and some cells become more susceptible to various pathological influences triggering the atherosclerotic process (Sobenin et al., 2013; Zorov et al., 2014).

Previous studies have revealed several atherosclerosis-associated mtDNA mutations, including m.3256C > T, m.3336T > C, m.5178C > A, m.12315G > A, m.14459G > A, m.15059G > A, and m.13513G > A. It was found that a certain spectrum of pro- and anti-atherogenic mtDNA mutations is characteristic for various types of atherosclerotic lesions of the human aortic intima (Sazonova et al., 2015).

As stated above, uncontrolled ROS production under stress of various origin changes the dynamics of mitochondrial functioning and increases mitochondrial fission and fragmentation at the expense of mitochondrial fusion. Apparently, nuclear respiratory factors (NRF1 and NRF2) play an important role in this process. These factors regulate the expression of the transcription factor of the mitochondrial genome (Tfam) and many other mitochondrial genes involved in oxidative phosphorylation. Recent studies demonstrated a link between mitochondrial dysfunction and insulin resistance through altered expression of the *PPARGC1A* gene in muscle and liver tissue. It was shown that the suppression of the synthesis of PGC1a protein (peroxisome proliferator-activated receptor gamma coactivator 1-alpha) impacted mitochondrial biogenesis and contributed to the induction of insulin resistance (Siasos et al., 2018). The function of PGC-1α is to stimulate mitochondrial biogenesis and promotes remodeling of muscle tissue into a fibrous composition and is also involved in the regulation of carbohydrate and lipid metabolism (Liang and Ward, 2006).

Study of the tRNA^Thr^ m.15927G> mutation revealed its association with coronary heart disease. The m.15927G > A mutation hindered the highly conserved base-pairing (28C-42G) of anticodon stem of tRNA^Thr^. Molecular modeling study demonstrated that the m.15927G > A mutation resulted in an unstable tRNA^Thr^ structure. The study conducted on cybrid lines bearing mitochondria with the m.15927G > A mutation showed a significant decrease in the efficiency of aminoacylation, which led to a decrease in the number of polypeptides encoded by mtDNA, respiratory failure, a decrease in membrane potential, and an increase of ROS production. The increased release of cytochrome c into the cytosol and caspase 3, 7, 9 and PARP proteins indicated that the presence of this mutation contributes to the development of apoptosis. These observations confirm the data published by different groups supporting the important effect of mitochondrial mutations on the pathophysiology of coronary heart disease (Jia et al., 2019).

Mitochondrial dynamics, fission, and fusion were first detected and studied in yeast. Over the past 10 years, it has become apparent that these processes are common to all cells containing mitochondria. Impairment of fission and fusion contributes to the disease development (Diot et al., 2016). The processes of mitochondrial dynamics determine the morphology of mitochondria, their qualitative and quantitative indicators, which, as it turned out, is critical for the development of cardiovascular diseases. These processes are related to the balance between energy requirements and nutrient intake. Changes in the morphology of mitochondria can be regarded as bioenergetic adaptation during pathological remodeling of cells of the cardiovascular system (Vásquez-Trincado et al., 2016). For example, aging processes are directly related to changes of mitochondrial dynamics. It was found that in old age, the volume, integrity, and functionality of mitochondria decreases due to the accumulation of mutations in mtDNA after oxidative damage caused by ROS. In elderly organisms, mitochondria are characterized by a decrease in the efficiency of oxidative phosphorylation, ATP production, an increase in the formation of ROS and a decrease in antioxidant protection. Moreover, the regulation of mitophagy and autophagy is impaired, preventing the removal of dysfunctional mitochondria. Similar processes enhance mitochondria-mediated apoptosis (Chistiakov et al., 2014).

It is likely that a large part of mtDNA mutations occurs due to the increased ROS production in the proximity of the mitochondrial genome. Accumulation of mtDNA mutations and impaired mitochondrial function contribute to atherogenesis, as has been confirmed by several studies. That, in turn, confirms the link between increased oxidative stress and cardiovascular risk (Yu and Bennett, 2014). However, oxidative stress cannot be considered as the main mechanism of mtDNA mutagenesis (Trifunovic et al., 2005; Itsara et al., 2014). The key factors in mtDNA mutation generation are replication errors by the mitochondrial DNA polymerase γ and spontaneous base hydrolysis (Kennedy et al., 2013).

Therefore, impaired mitochondrial function, biogenesis, and dynamics disrupt the cell homeostasis. Mitochondrial damage contributes to aging and a number of age-related pathologies. Thus, in the fight against aging and age-related diseases, it is possible to use strategies that effectively improve or eliminate defects in mitochondrial dynamics. To achieve this goal, it is necessary to develop small molecules capable of enhancing mitochondrial biogenesis and inducing mitophagy for patients with age-related disorders. Consequently, effective new therapeutic strategies should include coordinated induction of both mitophagy and mitochondrial biogenesis to maintain a healthy mitochondrial population. Further intervention studies are needed to test how mitophagy and mitochondrial compounds that induce biogenesis affect human physiology. Therefore, the knowledge of the mutation spectrum of the mitochondrial genome may be useful for attending physicians and medical geneticists for early detection of atherosclerosis and analysis of predisposition to its development.

## Role of Mitochondrial Mutations in Cellular Mechanism of Atherosclerosis; Chronification of Inflammation

Some of the above mentioned pro-atherogenic mtDNA mutations associated with atherosclerosis also correlated with pro-inflammatory activation of monocytes in primary culture (Orekhov et al., 2015). Two homoplasmic mutations, m.1811A > G and m.9477G > A, correlated with the degree of monocyte activation. At least three heteroplasmic mutations (m.14459G > A, m.1555A > G, and m.12315G > A), also correlated with pro-inﬂammatory activation of circulating human monocytes. Thus, some mutations may alter monocyte activation in atherosclerosis through mitochondrial dysfunction.

Studies on cybrid cell lines carrying variants of the mitochondrial genome obtained from atherosclerotic patients revealed the disturbances of mitochondrial functions including impaired mitophagy (Orekhov et al., 2019).

Mitophagy is involved in the innate immune response (Orekhov et al., 2020a). Inhibition of mitophagy in primary culture of human monocyte-derived macrophages increased lipopolysaccharide-induced pro-inflammatory response in the form of up-regulation of the IL-1β gene both in control cells and in the presence of mitophagy inhibitors. Repeated stimulation caused a much smaller pro-inflammatory response in control cells. When mitophagy was suppressed, re-stimulated cells continued to demonstrate a pro-inflammatory response. This means that suppression of mitophagy leads to a loss of immune tolerance and uncontrolled continuation of the pro-inflammatory response of macrophages.

**Figure 2:**
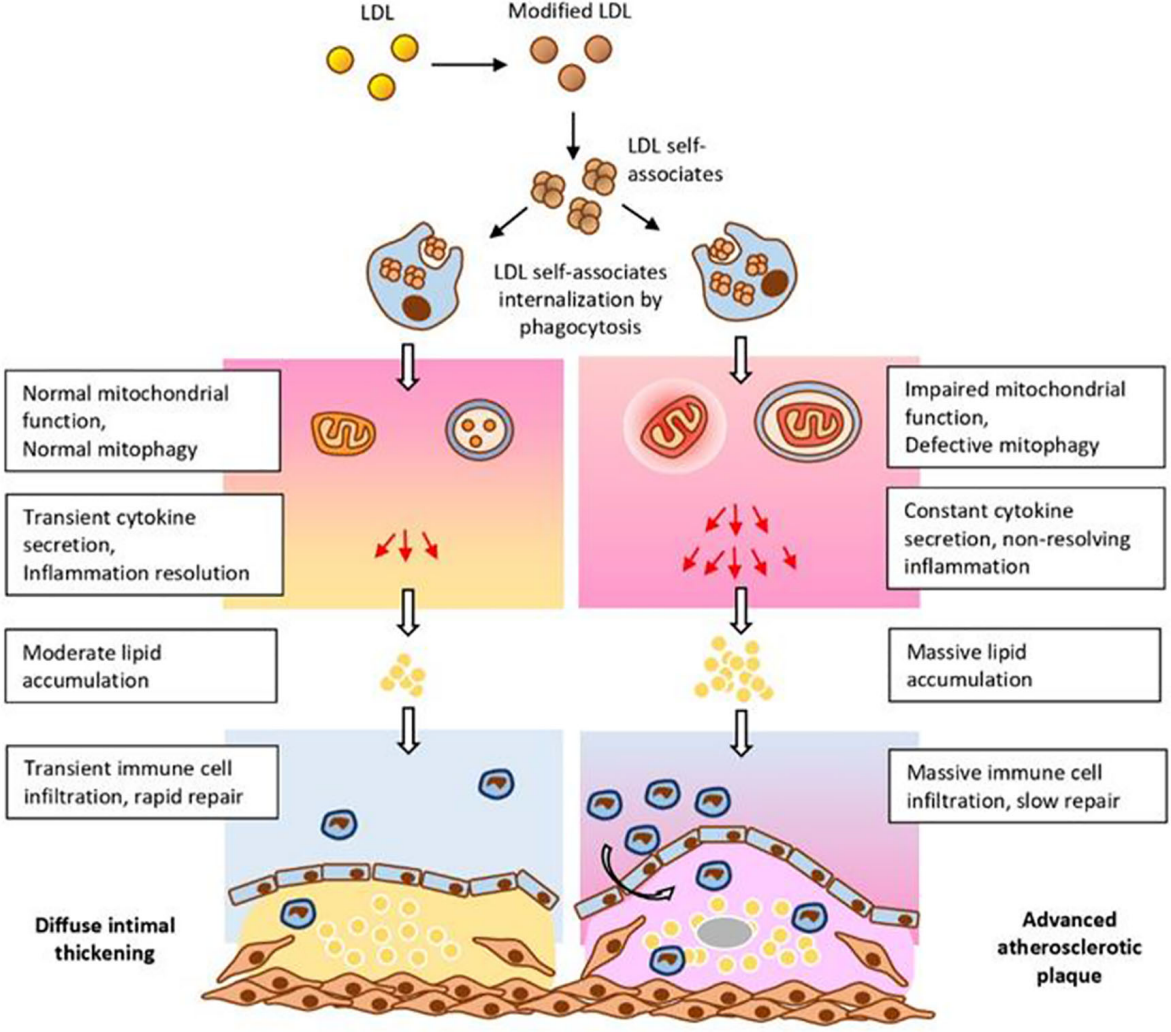
Impaired mitochondrial function and deficient mitophagy promote atherosclerotic lesion formation. Multiply modified LDL particles being accumulated and then internalized by macrophages are capable to alter mitochondrial function which ultimately leads to the formation of atherosclerotic plaques. From Orekhov et al. (2020a), with permission.

These and other data allowed to formulate a hypothesis explaining the important role of mitochondrial mutations in atherogenesis (**Figure 2**). According to this hypothesis, circulating atherogenic multiple modified LDL induces lipid accumulation in the arterial cells (Tertov et al., 1992). Modified LDL particles form self-associates taken up by arterial cell *via* nonspecific phagocytosis (Tertov et al., 1989). Stimulation of phagocytosis activates the pro-inflammatory response of macrophages (Orekhov et al., 2020b). This causes accumulation of intracellular lipids (**Table 1**). In normal response of innate immunity, the pro-inflammatory reaction resolves quickly. However, in the case of macrophages carrying mtDNA mutations associated with defective mitophagy “non-stop” pro-inflammatory response is produced that does not arrest. Local inflammation induced by this “non-stop” response becomes chronic atherosclerotic lesion is formed.

## Conclusions

The genetic aspects of atherosclerosis attract much attention in the current research. As novel genetic methods evolve, a huge amount of data becomes available, including identification of mtDNA mutations. While genomic mutations associated with atherosclerosis are relatively well studied, mtDNA mutations provide new opportunities for development of novel diagnostic and therapeutic tools. The discovered set of mutations in the mitochondrial genome has a clear connection with the likelihood of the development of the disease, its course, and prognosis. The acquired knowledge on the mitochondrial involvement in the development of atherosclerosis and chronic inflammation will be used for designing more selective, mitochondria-targeting treatments.

## Author Contributions

AM wrote the manuscript text. IS and DZ supervised the work and reviewed the manuscript. AG and AO conceptualized the work and obtained funding.

## Funding

This work was supported by the Russian Science Foundation (Grant # 19-15-00297).

## Conflict of Interest

The authors declare that the research was conducted in the absence of any commercial or financial relationships that could be construed as a potential conflict of interest.

## Abbreviations

bFGF, Basic fibroblast growth factor; CAD, Coronary artery disease; CVD, Cardiovascular disease; GWAS, Genome-wide associations study; HDL, High-density lipoprotein; IL, Interleukin; LDL, Low-density lipoprotein; LDLR, Low-density lipoprotein receptor; lncRNA, Long non-coding RNA; MCP-1, Monocyte chemoattractant factor 1; PDGF, Platelet-derived growth factor; RGN, Regulatory gene networks; ROS, Reactive oxygen species; SNP, Single nucleotide polymorphism; TNF, Tumor necrosis factor.
